# Supplementary material for: BMI‐1 modulation and trafficking during M phase in diffuse intrinsic pontine glioma
Source: FEBS Open Bio. 2025 Dec 27;16(6):1213–21. doi: 10.1002/2211-5463.70189 (PMC13238917; doi:10.1002/2211-5463.70189)
Supplement: Supplementary file 1 — Fig. S1. Further evidence indicating that BMI‐1 undergoes phosphorylation and translocation specifically during the M phase, as referenced in Fig. 1. Fig. S2. Additional evidence that BMI‐1 translocation during M phase is an active process, related to Fig. 2. Fig. S3. Depiction of the newly identified NES domain within BMI‐1 and Sanger sequencing analysis of BMI‐1 constructs bearing a truncated NES domain. [file FEB4-16-1213-s001.pdf]

Umaru, Figure S1

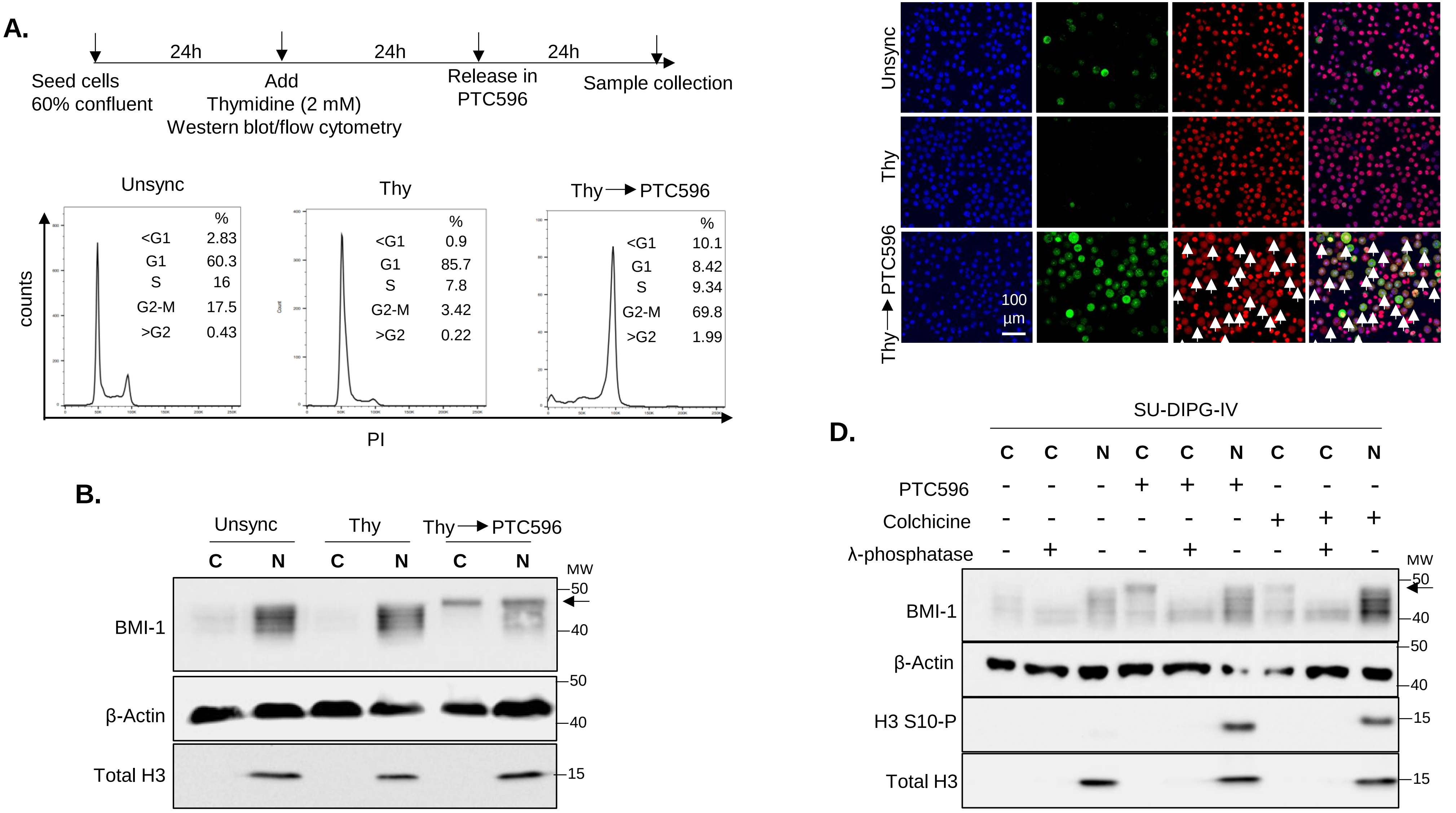

Figure S1: Further evidence indicating that BMI-1 undergoes phosphorylation and translocation specifically during the M phase, as referenced in Figure 1.

(A) Scheme representing the cell synchronization studies with thymidine block and release in PTC596, along with collection timepoints for cell cycle analysis (with indicated percentage of cells).

(B) Immunoblot analysis of BMI-1 from cytoplasmic (C) and nuclear (N) fractions. Actin and total H3 served as loading control. Arrow indicates phosphorylated BMI-1. MW indicates Molecular Weight marker in kDa.

(C) Representative immunofluorescence images of Cyclin B1 (green) and BMI-1(red). DAPI (blue) represent nuclei. White arrows indicate the cells with cytoplasmic localization of BMI-1. CCHMC-DIPG-1 cells were used for the above experiments. The scale bar is 100 µm.

(D) Immunoblot analysis of BMI-1 from cytoplasmic (C) and nuclear (N) fractions of SU-DIPG-IV cells treated with PTC596 (100 nM for 24h) or colchicine (100 ng/mL for 24 h). Cytoplasmic fractions were further treated with or without λ-phosphatase. Arrow indicated phosphorylated BMI-1. MW indicates Molecular Weight marker in kDa.

Umaru, Figure S2

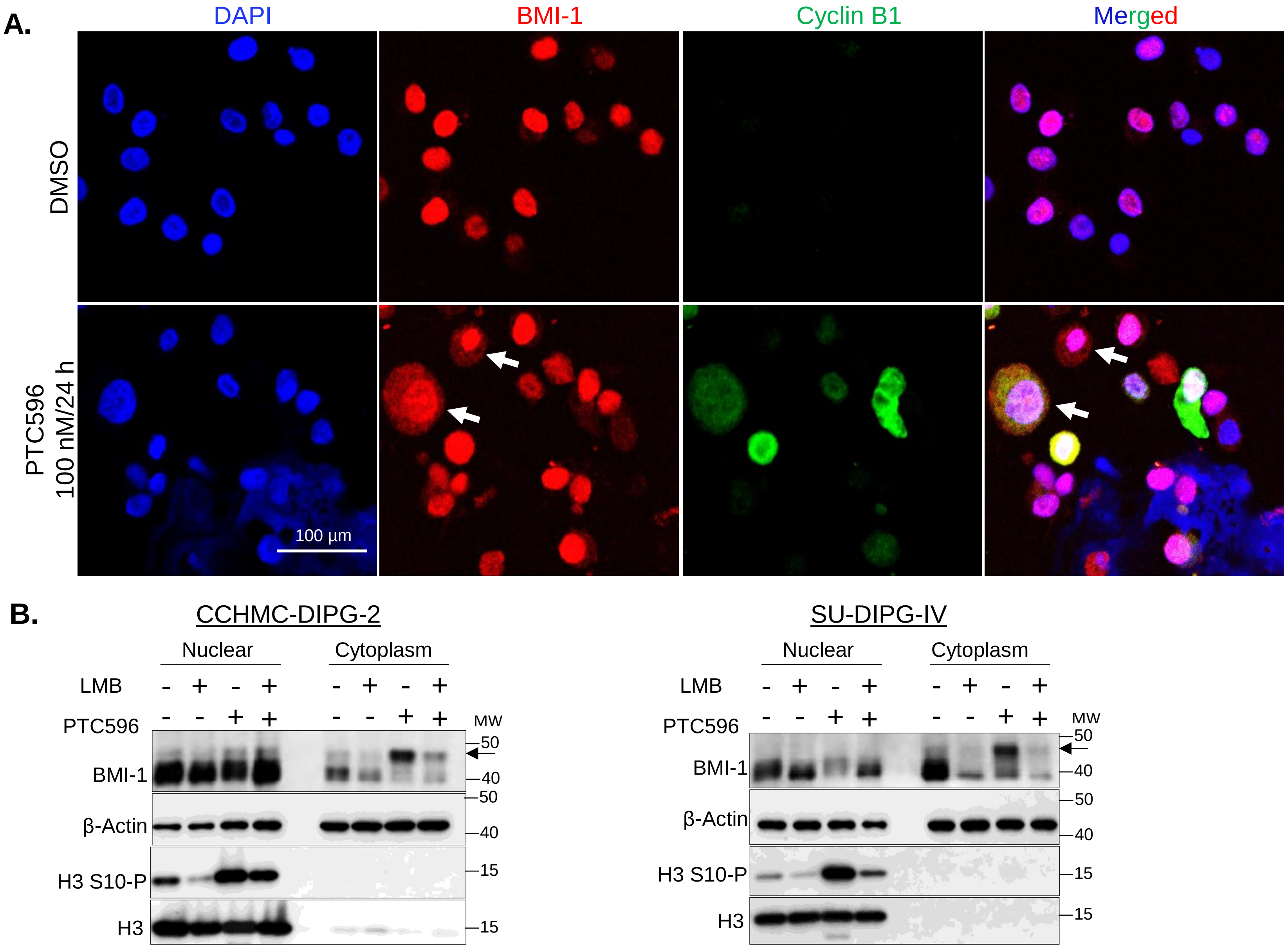

Figure S2: Additional evidence that BMI-1 translocation during M phase is an active process, related to Figure 2.

(A) Representative immunofluorescence images of BMI-1 (red) and Cyclin B1(green). White arrows indicate cells with both cytoplasmic and nuclear BMI-1. DAPI (blue) represents nuclei. The scale bar is 100 µm.

(B) Immunoblot analysis of BMI-1 and H3 S10-P in the cytoplasmic and nuclear fractions of CCHMC-DIPG-2(left) and SU-DIPG-IV (right) cells treated with LMB, PTC596, or both following the scheme in Figure 2B. β-Actin and total H3 served as loading controls for the immunoblot assay. Arrow indicates phosphorylated BMI-1. MW indicates Molecular Weight marker in kDa.

Umaru, Figure S3

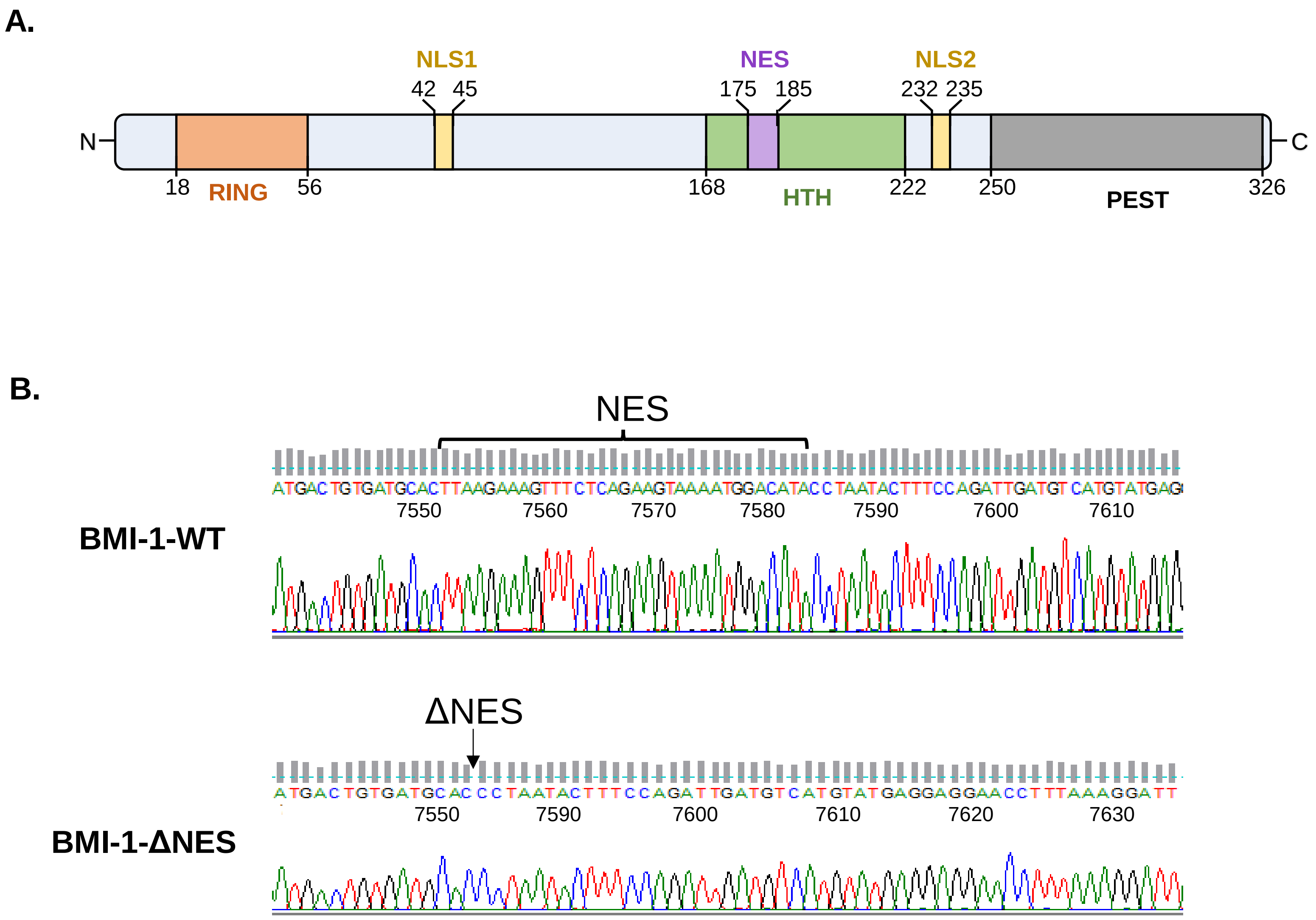

Figure S3: Depiction of the newly identified NES domain within BMI-1 and Sanger sequencing analysis of BMI-1 constructs bearing a truncated NES domain.

(A) Schematic illustration of different domains of the BMI-1 protein structure. The newly identified NES domain within the HTH domain is indicated.

(B) Genomic DNA extracted from D1-BMI-1 and D1-ΔNES-BMI-1 cells were PCR amplified, and Sanger sequenced to confirm deletion of the NES sequence. The numbers indicate the nucleotide position in *BMI-1* gene.
